# Diagnosing skin neglected tropical diseases with the aid of digital health tools: A scoping review

**DOI:** 10.1371/journal.pdig.0000629

**Published:** 2024-10-07

**Authors:** Ewelina Julia Barnowska, Anil Fastenau, Srilekha Penna, Ann-Kristin Bonkass, Sophie Stuetzle, Ricky Janssen

**Affiliations:** 1 Department of Health, Ethics & Society, Faculty of Health, Medicine and Life Sciences, Maastricht University, Maastricht, The Netherlands; 2 Department of Global Health, Institute of Public Health and Nursing Research, University of Bremen, Bremen, Germany; 3 German Leprosy and Tuberculosis Relief Association (DAHW), Wuerzburg, Germany; 4 Marie Adelaide Leprosy Center, Karachi, Pakistan; 5 Heidelberg Institute of Global Health, University of Heidelberg, Heidelberg, Germany; 6 Department of Health, Ethics & Society, Care and Public Health Research Institute (CAPHRI)/MHeNs School for Mental Health and Neuroscience, Faculty of Health, Medicine and Life Sciences, Maastricht University, Maastricht, The Netherlands; Instituto Politécnico Nacional Escuela Superior de Medicina: Instituto Politecnico Nacional Escuela Superior de Medicina, MEXICO

## Abstract

Delays in diagnosis and detection of skin neglected tropical diseases (NTDs) pose obstacles to prompt treatment, which is crucial in preventing disability. Recent developments in digital health have given rise to approaches that could increase access to diagnosis in resource-poor areas affected by skin NTDs. This scoping review provides an overview of current digital health approaches that aim to aid in the diagnosis of skin NTDs and provides an insight into the diverse functionalities of current digital health tools, their feasibility, usability, and the current gaps in research around these digital health approaches. This scoping review included a comprehensive literature search on PubMed, EMBASE and SCOPUS, following the PRISMA guidelines. Eleven studies were included in the review and were analysed using a descriptive thematic approach. Most digital tools were found to be mobile-phone based, such as mobile Health (mHealth) apps, store-and-forward tele-dermatology, and Short Messaging Service (SMS) text-messaging. Other digital approaches were based on computer software, such as tele-dermatopathology, computer-based telemedicine, and real-time tele-dermatology. Digital health tools commonly facilitated provider-provider interactions, which helped support diagnoses of skin NTDs at the community level. Articles which focused on end-user user experience reported that users appreciated the usefulness and convenience of these digital tools. However, the results emphasized the existing lack of data regarding the diagnostic precision of these tools, and highlighted various hurdles to their effective implementation, including insufficient infrastructure, data security issues and low adherence to the routine use of digital health tools. Digital health tools can help ascertain diagnosis of skin NTDs through remote review or consultations with patients, and support health providers in the diagnostic process. However, further research is required to address the data security issues associated with digital health tools. Developers should consider adapting digital health tools to diverse socio-cultural and technical environments, where skin NTDs are endemic. Researchers are encouraged to assess the diagnostic accuracy of digital health tools and conduct further qualitative studies to inform end-user experience. Overall, future studies should consider expanding the geographical and disease scope of research on digital health tools which aid the diagnosis of skin NTDs.

## Author summary

Skin Neglected Tropical Diseases (NTDs) consist of a group of potentially severely disabling conditions, such as buruli ulcer, leishmaniasis, leprosy, lymphatic filariasis, mycetoma, scabies and other ectoparasitoses, yaws, and onchocerciasis. Despite the severity of these conditions, access to diagnosis and treatment for skin NTDs faces significant constraints in remote and resource-deprived areas. The scarcity of dermatological expertise and the lack of adequate medical facilities in these settings contributes to challenges in effectively addressing these conditions. Currently, there are various studies that describe the role of telemedicine and mHealth and their potential in control and management of skin NTDs in endemic regions. Yet, there is a current lack of comprehensive analysis and evidence synthesised concerning the utilization of a range of digital health tools to improve access to diagnosis of skin NTDs and the user experience of these tools in practice. Using a scoping review approach, we have synthesised the available evidence and user-experience on the range of digital health tools that aim to aid in the diagnosis of skin NTDs in underserved areas.

Overall, the findings of this scoping review have highlighted the benefits of portable digital health tools which can be used to facilitate consultations with patients directly in the community, thereby extending dermatological healthcare to remote and resource-limited areas. However, our study highlights the current lack of data on the diagnostic accuracy of digital health tools, as well as several challenges to safe and successful implementation, such as inadequate infrastructure, data security issues and low adherence to the routine use of digital health tools. In conclusion, we offer developers and policymakers recommendations for the future development of digital health tools designed to enhance the diagnosis of skin NTDs, along with potential avenues for future research.

## Introduction

Skin Neglected Tropical Diseases (NTDs) represent the most common infections of people living in poverty in sub-Saharan Africa, South Asia, Southeast Asia, Latin America, and the Caribbean [1]. The associated skin manifestations of NTDs exert a significant amount of psychological, physical, and social burden on individuals, with an estimated impact on more than one billion people in the world [2]. NTDs are often found to be co-endemic, whereby an individual may suffer multiple conditions at once, causing severe pain, disability, and neurological problems, which can last for decades [3]. Skin NTDs consist of a group of cutaneous preventable conditions, which are major contributors to disability-adjusted life years [DALYs] among some of the world’s most marginalised communities. There are over ten cutaneous diseases belonging to NTDs, as prioritised by the WHO, which include buruli ulcer, leishmaniasis, leprosy, lymphatic filariasis, mycetoma, scabies and other ectoparasitoses, yaws, and onchocerciasis [4].

Despite the existing frameworks for tackling NTDs in the global health agenda, skin NTDs continue to be under-diagnosed in populations living in remote and poor settings around the globe, where there is a lack of resources or expertise for correct diagnosis, and where treatment is unavailable or inaccessible [5]. Estimates have shown that nearly every person in the world’s poorest ‘bottom billion’ has suffered at least one NTD [5]. Most importantly, the associated morbidity and mortality inflicted upon individuals are mostly preventable [6].

Skin NTDs are often underreported and have been more neglected compared to other NTDs, which have been targeted by mass drug administration and surveillance programs [7]. For skin NTDs such as mycetoma or cutaneous leishmaniasis, the lack of awareness among health workers leads to delayed diagnosis and treatment [8]. Low reporting of skin NTD cases within endemic countries creates difficulties in the assessment of the overall burden of disease, particularly when projects and interventions are limited in their geographical scope [9].

When provided with the necessary resources and expert guidance, any health worker can work to more effectively recognise signs of skin NTDs [10]. Utilising the capabilities of peripheral or community health workers present on the ground can be useful in low- and middle-income countries (LMICs), where there is a severe shortage of dermatologists and specialists, and where most physicians are concentrated in urban areas with few available in rural regions [7]. Therefore, there is a need for capacity building interventions and implementation of long-distance tools to provide support for workers in remote areas to increase the access to quality dermatology care and diagnostics [11].

The significant commonalities and co-endemicity of several skin neglected tropical diseases (NTDs) present an opportunity for developing integrated interventions. These integrated approaches are crucial for alleviating the disease burden associated with skin NTDs. Integration, in this context, involves the strategic grouping of multiple diseases to facilitate joint interventions through a common platform. This unified approach offers several benefits, such as enhancing the efficiency of detection and diagnosis. By leveraging common tools, technologies, and the health workforce across different skin NTDs, health programs can achieve more with the same resources. This is especially important in resource-limited settings where healthcare resources are often scarce [4].

In recent years, the WHO has recognised that digital health tools can play a crucial role in global healthcare delivery for skin NTDs and offer substantial support to health workers in the community. The WHO’s recently developed mobile application, ’SkinApp,’ assists frontline health workers in diagnosing and managing skin NTDs. Using an algorithm, it helps identify the signs and symptoms of several skin NTDs. In this way, digital health solutions may improve the early detection and management of skin NTDs and contribute towards alleviating the morbidity and mortality associated with these conditions [12].

Existing literature reviews have described eHealth and tele-dermatology as cost effective and time efficient tools in the control and management of skin NTDs and represent a hopeful paradigm of delivering remote dermatological expertise and care [13]. There are different types of eHealth strategies available aiding the diagnosis and case notification of skin diseases in resource limited settings [14]. These include tools such as mHealth, telemedicine, video conferencing technology, email, or secure mobile messaging services, among other platforms, which have been reported to improve access to health care for rural and resource-poor populations [15]. However, the recent increase in unregulated mobile health apps has raised important concerns, which include data privacy, limited technical requirements or poor data quality [16]. Studies show that developers of digital health tools can improve how these technologies are perceived and utilised by end-users, by employing more user-centred design processes [17].

Existing literature reviews have been able to describe both mHealth [16] and tele-dermatology interventions [12] aimed towards controlling skin NTDs. However, none of the existing literature has specifically examined the role of digital tools in aiding the diagnosis of skin NTDs in endemic areas. Existing reviews have limited their scope to solely mHealth or telemedicine and focused on describing the general utility of the respective intervention in contributing to the epidemiological and clinical management of skin NTDs.

Therefore, this paper aims to map the available evidence on the currently available digital health tools which can be used to facilitate the diagnosis of skin NTDs, which will be beneficial for both developers and NTD health experts, when considering the implementation of this technology for skin NTDs. The main research questions were as follows.

1. What are the various digital tools that facilitate the diagnosis and detection of skin NTDs?
  1.1. What are the specific features of these tools, and how do they differ?
2. What is the impact of implementing these digital health tools for diagnosing skin NTDs, especially in low-resource settings?
  2.1. What are the perceptions and experiences of end-users regarding these digital tools?
  2.2. What are the benefits and challenges associated with the implementation of these digital health tools?
3. What recommendations should be considered for the future development and implementation of digital tools for diagnosing and detecting skin NTDs?

To address these research objectives, a scoping review was conducted. A total of 369 articles were screened for eligibility, with 11 undergoing thematic analysis. The methodology is further described in the following section. The authors utilized established mHealth [18] and telemedicine [19] taxonomy models to develop relevant themes which were as follows: technical modality, function and application, policy considerations, and end-user factors. Based on the findings of this review, several recommendations are proposed for the future development and implementation of digital health tools for diagnosing skin NTDs.

## Methodology

The purpose of this study was to map the available evidence related to different digital health tools used to aid the diagnosis of a range of skin NTDs. A scoping review approach was adopted to explore this broad topic and provide insight into the main characteristics and concepts within available sources of evidence [20]. Given that the concept of diagnosing skin NTDs using digital approaches is relatively new, this method offers a comprehensive overview of the current landscape of digital health solutions and identifies areas for further research in the field of skin NTDs [21].

Firstly, we outline our methods for conducting the scoping review, following the PRISMA-ScR guidelines and PRESS checklist for search strategies (S1 Checklist) [22,23]. We provide details on the search strategy, selection of studies, as well as data collection and analysis methods. Following this, we present the results of the scoping review, including an overview of the study characteristics of the included publications and the findings from thematic analysis. We then discuss the implications of these results for future implementation of digital health tools in this field and conclude with recommendations for developers, policymakers and future studies. A protocol was not registered for this review.

### Search strategy

A comprehensive literature search was conducted in MEDLINE (Pubmed), EMBASE (Ovid), and SCOPUS databases on July 24^th^, 2023. Firstly, a preliminary literature search was performed to identify medical subject heading (MeSH) terms and key words. These terms were organized into three main concepts: skin neglected tropical diseases AND diagnosis AND digital health tools. These concepts formed the structure of the search string on PubMed and were then translated into Embase and SCOPUS databases. The full search strategy is provided in S1 Table and includes the number of results returned on each database. Additionally, manual handsearching of citation indexes was performed to identify any additional studies.

The selected combination of databases was chosen to encompass a broad spectrum of journals and literature types, thereby maximizing the retrieval of relevant studies. PubMed is dedicated to health and medicine, making it an essential resource for health-related scoping reviews [24]. Embase offers a wider array of international journals compared to PubMed, providing a more global perspective [25]. Scopus is one of the largest abstract and citation databases of peer-reviewed literature, and its multidisciplinary nature helps capture a broad spectrum of relevant studies [26]. Together, these databases covered a wide range of disciplines such as health sciences and social sciences, which aided in answering the research questions posed by the review.

### Selection criteria

The review included studies published in the last 10 years, up to July 24^th^, 2023. Primary research, not restricted to any study design, language, and geographic setting, was included. Other sources such as conference papers, abstracts, preprints, and letters were considered and screened based on inclusion criteria. The review included studies that reported on all of the following three elements:

1. Patients, health providers or healthcare organisations that were seeking, or currently working with, digital health tools for facilitating the diagnosis of at least one skin NTD. Skin NTDs were defined as any of the cutaneous diseases as prioritised by the WHO, which include Buruli ulcer; cutaneous leishmaniasis; leprosy; lymphatic filariasis; mycetoma, chromoblastomycosis and other deep mycoses, including sporotrichosis; onchocerciasis; post-kala-azar dermal leishmaniasis; scabies and other ectoparasitoses, including tungiasis; and yaws.
2. Any ehealth, mhealth or telemedicine intervention which involved the use of technology to facilitate a patient-provider or provider-provider interaction, and support a remote consultation, related to the patient’s diagnosis.
3. Evidence on how the digital tools were able to facilitate the diagnosis of skin NTDs, exploring aspects such as usability, applicability, feasibility, and user experience.

Secondary research such as reviews, meta-analysis and study protocols were excluded, as well as studies solely focusing on outcomes such as education, skin NTD prevention or treatment.

### Data collection and analysis

The combined search results from all databases were uploaded to EndNote 20 reference manager to remove duplicates. Following this, the references were transferred on to Rayyan Software and screened for suitability based on the content of titles and abstracts and their alignment with the inclusion criteria. Next, a full text review was conducted by two authors and screened using previously established inclusion and exclusion criteria. The data from the resultant articles was extracted according to the following study characteristics: name of author, year of publication, study design and population, skin NTD, type of digital tool, aims, interventions and findings. The data extraction table (Table 1) and supporting information documents (S2 Table and S3 Table) were synthesised in accordance with the research questions, to facilitate comparison and analysis.

**Table 1:** Characteristics of included studies.

^a^Author, Publication Type	Study Design	Study Population/ Setting	Skin ^k^NTD	Digital tool	Aim	Intervention	Key findings
Micheletti, R. G. (2014) Research Letter	Retrospective chart study	Gaborone, Botswana Urban area LMIC Patient population N = 300	^i^SCB ^b^LEP	Robotic tele-dermatology	To guide the impact of the system in facilitating the diagnosis of skin diseases	Cases were submitted for teleconsultation via the robotic microscope over a 2.5-year period.	• In over 80% of cases, tele-dermatopathology contributed to the clinical management of skin disease by facilitating diagnoses and guiding treatment through remote review. • A robotic microscope operated by a dermatopathologist confirmed 43 diagnoses that were not initially picked up by the doctor in clinic.
Villalon, E. E. (2015) Conference abstract	Pilot study +qualitative feedback	Iloilo City and 19 [of 42] municipalities in Iloilo, Philippines. Rural and urban areas LMIC	^b^LEP	Leprosy alert response network and surveillance system [mobile phone-based teleconsultation system]	To describe the implementation of LEARNS in Iloilo to reduce delays in diagnosis and treatment.	The providers used LEARNS to send an image of the skin lesion and patient information through a mobile phone to a specialist for diagnosis.	• Of the 8 leprosy cases, 5 were detected through LEARNS in 2014. • The maintenance cost of LEARNS is low, as SMS can be used to send images. • LEARNS can complement leprosy control and monitoring programmes, as well as increase capacity of community health workers in diagnosing leprosy at the community level.
Mieras, L. F. (2018) Short communiation article	Pilot study [+qualitative semi structured interviews and focus groups]	Zambezia Province [1^st^ version], Nampula Province [2^nd^ version] Mozambique Rural areas, LMIC	^b^LEP ^c^BU ^d^CL ^e^LF ^f^MYC ^g^ONC ^h^PDC ^i^SCB ^j^YS	[NLR] Skin App	To develop an app to support peripheral health workers in the diagnosis of several skin diseases in resource poor areas.	The SkinApp developed a mobile-based algorithm for the diagnosis and management of skin ^k^NTDs [included WhatsApp function]. User feedback was obtained.	• Smartphone ownership among health workers in Zambezia is high, which suggests that the use of SkinApp is a realistic option. • The app supported health workers in ascertaining diagnoses and guiding treatment of skin diseases at the peripheral level. • The performance of the app as a diagnostic tool was not studied.
Messagier, A. L (2019) Research article	Retrospective chart review [+satisfaction survey]	French Guiana Rural areas Patient population N = 254	^i^SCB ^b^LEP ^d^CL	Tele-dermatology system	To assess the quality of the telemedicine dermatology service and evaluate its usefulness in facilitating diagnosis	Health providers in delocalised centres referred cases to remote dermatologists. Data was collected from the requests and a user satisfaction survey was conducted.	• Diagnostic concordance was found to be 55%. • The accuracy of the diagnosis was found to increase with the quality of the images provided [however image quality was ‘good’ in only 60% of cases]. • The Guyanese tele-dermatology system is an efficient, reliable, and accessible alternative to face-to-face health care delivery in areas lacking dermatological expertise.
Lee, C.H. (2020) Research article	Pilot study [+patient satisfaction survey]	Taiwan District hospital in Taitung County Rural areas	^i^SCB	Real-time and face-to-face tele-dermatology consultation [Intouch lite software]	To establish a tele-dermatology consultation service in Taiwan and improve access to care	Teleconsultations were carried out via the Intouch lite software. Patient satisfaction with the service was sought via questionnaire.	• Skin diseases, such as scabies, could be easily and promptly diagnosed using tele-dermatology and dermoscopic images, due to the distinct and diagnosable features of the disease. • Patient Satisfaction rate was found to be over 80% annually. No further detail regarding what determined ‘patient satisfaction’ was given.
Ferreira da Silva, P. E. (2020). Research article	Pilot study [+semi structured questionnaire]	Brazil [Montes Claros, Paracatu, Lavras municipalities] Endemic areas, LMIC	^l^VTL	LeishCare app	To describe LeishCare, an application designed to aid the diagnosis and management of leishmaniasis	The app was used by health providers as an aid in the diagnosis and management of patients with suspected leishmaniasis. User perception was evaluated.	• In the 12-month evaluation, 95% of users reported that the app fulfilled or positively exceeded their expectations. The app may enable early diagnosis and improved management of ^l^VTL by clarifying uncertainties in diagnosis or treatment. • The study reports low adherence to the routine use of the app among health providers.
Rubiano, L. (2021) Research article	Prospective cohort study [+Qualitative questionnaire]	Colombia–Tumaco and three rural areas [La Guayacana, Llorente and El Pinde] within the Tumaco municipality Rural areas, LMIC Patient population N = 122	^d^CL	Guaral/Leishmaniasis app	To evaluate the performance and usability of a mobile application for diagnosis and referral of leishmaniasis	Community healthcare workers applied the clinical prediction rule in the diagnostic process using the mobile app and referred suspected ^d^CL cases for further evaluation by a remote physician.	The sensitivity of the app was very high [>95%] in detecting true ^d^CL cases. • Positive responses in feedback questionnaire such as ‘it was easy to identify ulcers/grouped lesions’, or ‘allows for rapid identification of CL lesions.’ The app can aid community health volunteers in active case detection of ^d^CL in rural areas, decreasing the interval between symptom onset and diagnosis.
Handa, S. (2021) Research article	Retrospective chart review [+patient satisfaction, physician feedback surveys]	North India Tertiary care centre [urban area] LMIC Patient population N = 6125	^b^LEP	Hybrid model telemedicine service	To provide an insight into the tele-dermatology service and its acceptability	Tele-dermatology consultations were provided during the COVID-19 pandemic. End-user feedback from physicians and patients was obtained.	• A definitive diagnosis was obtained in 93% of patients who used tele-dermatology services. • Patients as far as 2000km away from the institution were able to access the tele-dermatology service. • Only 6125 out of 7530 possible tele consults were successfully carried out due to technical barriers, for ex. connectivity and internet issues.
Parajuli, N. (2023) Research article	Case Series	Kalikot, Nepal Rural areas, LMIC	^d^CL	Mobile Tele-dermatology–Viber mobile app	To demonstrate how the mobile app Viber, was used to facilitate diagnosis and management	Photo consultations with a dermatologist at a central level were carried out through done the Viber mobile application	• The Viber mobile app enabled case discussions with specialists and helped ascertain the probable diagnoses of cutaneous leishmaniasis in rural areas.
Yotsu, R. R. (2023b) Research article	Mixed Methods Pilot Study	Sinfra Health District, Côte d’Ivoire Rural areas, LMIC Patient population N = 207	^b^LEP ^c^BU ^e^LF ^i^SCB ^j^YS	eSkinHealth app	To analyse the usability and effectiveness of the eSkinHealth app for diagnosis and management of several skin diseases	The usability of the app was evaluated through the System Usability Scale [SUS] and in-depth interviews. The effectiveness was determined by its ability to detect and manage several skin ^k^NTDs.	• A total of 79 cases of skin ^k^NTDs were reported in those who received the eSkinHealth app as compared to 17 cases in the control. • SUS scores increased during the intervention–showing that the time needed to get accustomed with the intervention was 2–3 months. • True effectiveness of the app could not be determined at the time of the study due to inaccurate data in the control arm.
Verma, N. (2023) Research article	Randomized crossover trial Diagnostic concordance study	Morbi District, Gujarat, India Rural areas, LMIC Patient population N = 104	^i^SCB	MyTeleDoc app	To assess the diagnostic concordance between remote telemedicine consultations to those of face-to-face care	Medical records from the teleconsultation [MyTeleDoc app] and face-to-face consultation were compared.	• In summary, 74% diagnostic concordance was observed between face-to-face and telemedicine consultations. However, diagnostic concordance within tele-dermatology was only 63%. • The study found that rural patients would be more likely to face geographic/ financial barriers—however, the availability of local primary health care centres which utilise the digital assistance service would help ease these challenges.

^a^Studies are displayed in chronological order, then alphabetical order of 1st Author

^b^LEP = Leprosy

^c^BU = Buruli Ulcer

^d^CL = Cutaneous Leishmaniasis

^e^LF = Lymphatic Filariasis

^f^MYC = Mycetoma

^g^ONC = Onchocerciasis

^h^PDC = Podoconiosis

^i^SCB = Scabies

^j^YS = Yaws

^k^NTD = Neglected Tropical Disease

^l^VTL = Visceral and tegumentary leishmaniasis [cutaneous and muco-cutaneous leishmaniasis

Subsequently, a descriptive thematic analysis was conducted on the included studies. A deductive approach was used to synthesize themes around the key features of digital health tools. In order to facilitate this, the authors utilized established mHealth [18] and telemedicine taxonomies [19], which discuss various dimensions of digital health tools such as "technology" and relevant sub-dimensions such as "synchronicity" and "network" or "connectivity”. The dimensions were organized into themes, which were constructed as the following: technical modalities, function and application, policy considerations and end-user experience. Data from the included studies was extracted according to these themes, involving discussions between the author and another co-author to ensure consistency in the thematic analysis. The results were consequently tabulated to ease interpretation and analysis (S2 Table and S3 Table). Following this, the data on user-experience was analysed using an inductive approach, and the emerging themes were identified as follows: usability, applicability, and feasibility.

This review used stringent inclusion criteria to ensure that the most pertinent studies were selected. It focuses on a highly specific area relating to skin NTDs and an emerging area within digital health tools, leading to a smaller pool of relevant studies [21]. Furthermore, narrowing the scope of this review to address the specific ‘diagnostic’ role of the digital health tools may have limited the number of available studies, but enhanced the depth and relevance of the analysis.

### Ethical approval

This study obtained ethical approval from the Global Health Programme Ethical Review Committee at Maastricht University.

## Results

### Selection of studies

**Fig 1:**
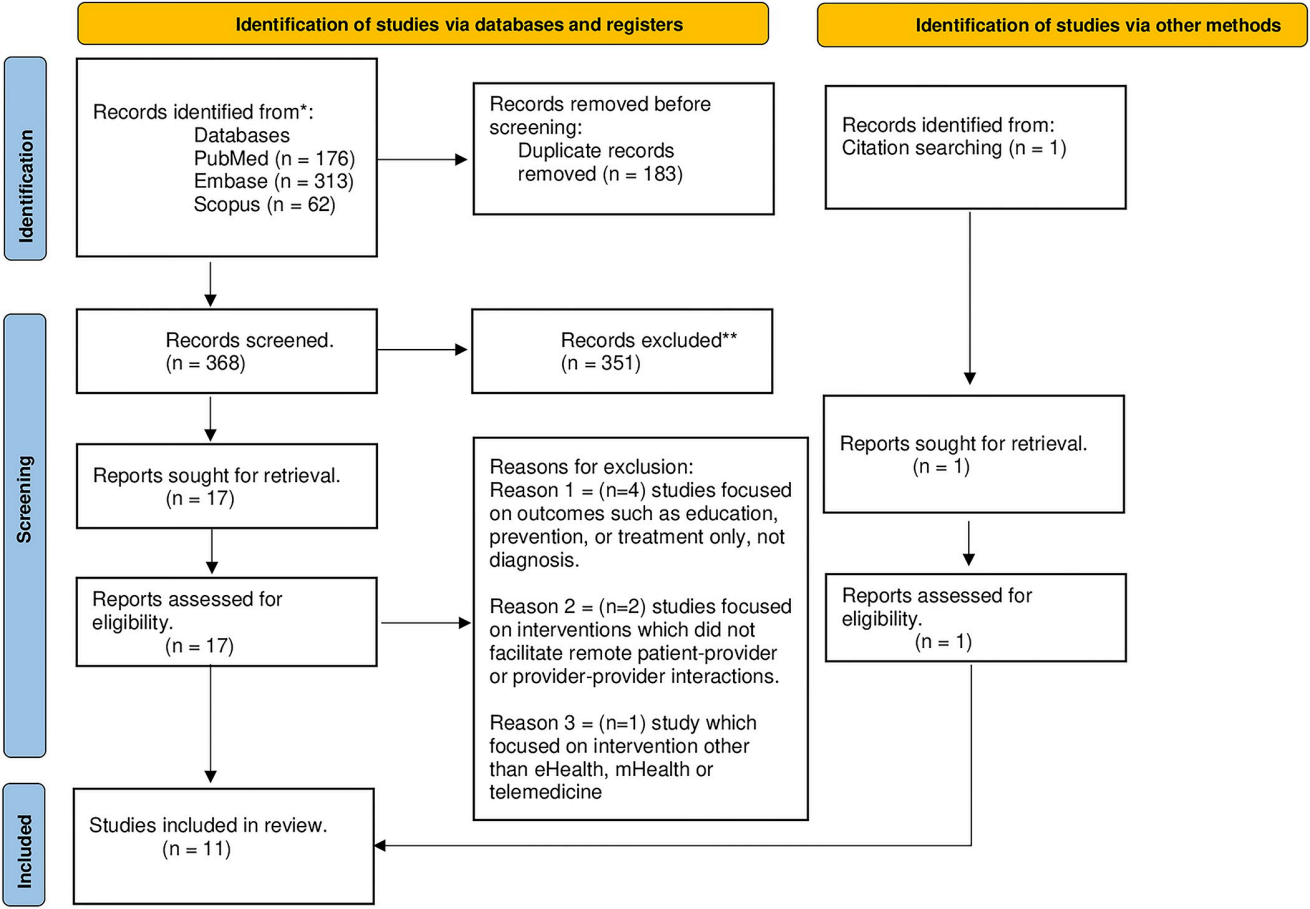
Flowchart of included studies.

The study selection process is reported in the flow diagram above (Fig 1) according to the PRISMA guidelines [27]. A total of 369 relevant articles were eligible for screening after removing duplicates. After screening of abstracts and titles, 18 out of 369 (4.9%) relevant publications were selected for full-text review. The 18 articles were screened for eligibility based on the inclusion and exclusion criteria, whereby seven (39%) did not meet the inclusion criteria and were excluded. Therefore, a total of eleven articles were included in this scoping review.

### Summary of study characteristics

The characteristics of the included studies are shown in Table 1. All studies reported on at least one skin NTD. Two studies reported on five or more skin NTDs; one study [28] included buruli ulcer, leprosy, lymphatic filariasis, scabies, and yaws, while another study additionally included cutaneous leishmaniasis, mycetoma, onchocerciasis, and podoconiosis [29]. Most studies (n = 8) reported on leprosy, scabies, or cutaneous leishmaniasis, alone or in combination. One study [30] focused on visceral and tegumentary (mucous/cutaneous) leishmaniasis. Most studies (n = 9) were conducted in LMICs. The studies were conducted in the ten following countries: Botswana (n = 1), Philippines (n = 1), Colombia (n = 1), Mozambique (n = 1), French Guiana (n = 1), Brazil (n = 1), India (n = 2), Taiwan (n = 1), Côte d’Ivoire (n = 1) and Nepal (n = 1).

There were eleven different digital health tools identified in total. The organisation of results was based on the thematic analysis and data was grouped into the following: technical modality, function and application, policy considerations, and user experience (with key themes such as usability, applicability, and feasibility).

### Technical modality

The technical modality of the digital health tools in this section refers to their specific technological features and components, such as the device type, synchronicity, connectivity, or operating systems. Most digital tools (n = 8) were found to be mobile phone based. Six of these mobile-based digital tools were mobile applications [28–33] and the other mobile-based interventions were focused on hybrid store-and-forward and real-time tele-dermatology [34] or sending images of suspected skin NTD lesions with brief clinical information through SMS between health workers for diagnostic support [35]. The use of SMS in resource-limited settings was beneficial for peripheral health workers who could send images and text messages at inexpensive communication charges, with no internet connection required. One pilot study [29] found that smartphone ownership among health workers in Zambezia, Mozambique, was very common. Furthermore, the widespread availability of the Android operating system on locally available and relatively inexpensive smartphones made mobile applications more suitable to be used in resource-limited settings, as opposed to iOS-based systems.

In addition to smartphone-based mobile applications, other digital tools included telemedicine computer software, such as tele-dermatopathology software with email store-and-forward [36]; computer-based telemedicine software with a digital camera [37]; and real-time, face-to-face tele-dermatology through Intouch lite software [38]. Most digital health tools (64%) required an internet connection to access their main functions [36]. Digital tools such as the Skin App [29], Guaral App [31], or eSkinHealth App [28] stored information collected on the app offline, which could then be synchronised via a database server on to a web-based platform once internet connectivity was re-established. As one study in Colombia highlighted, network, and internet accessibility can be very limited in some rural areas [31], therefore an offline function can be beneficial in these settings.

Digital tools, which utilised online store-and-forward tele-dermatology, have reported to use social media mobile applications such as WhatsApp [34] and Viber [32]. The use of these social media apps helped health workers to send supplementary photographs of skin lesions and clinical information to remote doctors to aid the diagnostic process. These apps were used as they were convenient and user-friendly platforms, widely used by the public [34]. However, one of the studies suggests that data privacy breach risks can pose a significant barrier to using mobile applications such as WhatsApp for tele-dermatology, particularly in contexts where data security laws are not rigorously enforced [34].

Another study describes an innovative feature of real-time tele-dermatology, whereby a hand-held diagnostic USB device (dermoscope) was connected to a PC/computer and enabled the doctor to view patient’s skin in 2M pixel detail, which in turn helped remote doctors ascertain a skin NTD diagnosis [38]. The study reported that the dermoscope enabled the visualisation of skin burrowing and scaling which helped the doctor diagnose scabies during the virtual consultation [38]. In summary, this section outlined the different technical modalities of current digital health tools and highlighted their potential in facilitating the diagnosis of skin NTDs.

### Function and application

In this section, the function and application of these digital health tools refers to their intended purpose, role, and practical use. All eleven digital health tools in the included studies were developed to aid in the diagnosis of skin NTDs. Most digital tools helped ascertain diagnosis by facilitating provider-to-provider tele-dermatology (n = 6), commonly between peripheral/community health workers and remote doctors [30–33,35,37]. Through this method, peripheral workers and non-specialists were able to confirm probable diagnoses remotely with dermatologists, enabling the patient to be managed accordingly. Other digital health tools (n = 3) facilitate tele-dermatology between patients and providers, such as real-time video teleconsultations [37], or through audio/video consults combined with supplementary photographs of skin lesions to aid in the diagnostic process [32,33].

Digital tools such as SkinApp [29] and the Guaral App [31] utilised an algorithm that guided health workers through the diagnostic process at the peripheral level, by providing instructions at each step. At the same time, the app enabled them to contact remote specialists for assistance. Other useful functions of the digital tools were found to be monitoring and follow-up capabilities [28,30,33,38], referral [31,32,37] and mentoring [29]. However, there was a lack of information found on the diagnostic accuracy of the digital tools in facilitating diagnosis of skin NTDs. Out of the included studies in this review, only one study measured the sensitivity of the Guaral App in detecting true leishmaniasis cases, but other important characteristics such as negative predictive value and specificity were not assessed [31].

Certain digital health tools attempted to address aspects of inclusivity in their digital tool design. This was emphasised by the developers of the eSkinHealth app who adapted the tool for use by people with skin types IV and darker, which aims to account for the range of skin colours in regions affected by skin NTDs [28]. Moreover, the SkinApp [29] is currently working on translating the mobile application into multiple languages to accommodate for a diverse target audience.

### Policy considerations

In this section, the policy considerations of digital health tools refer to issues surrounding privacy, security, and data management, which impact the safe use of digital health tools. Most studies (73%) did not address any potential data privacy or management concerns. In one study, the authors expressed that in a country such as India there is limited guidance on the duration of data storage, and the responsibility of data management lies at the hands of the physician [34]. Only a few of the studies reported on privacy and security measures of the digital health tools. For example, one digital tool, LEARNS, employed standardised and de-identified patient information in SMS texts between health workers to protect confidentiality [35]. The telemedicine computer software in French Guiana utilised a secure network that is appropriate for the sharing of confidential medical data [37]. Furthermore, the Guaral App [31] utilised strong encryption, and two-factor authentication is required to gain access to the app, which protects patient information. Another mobile application, eSkinHealth app, maintained patient information security using QR codes, and stored data on an encrypted and regulated server (Simple Storage Service–Amazon Web Service Server) with 24-hour back-up. Access to the patient data was regulated through user levels to maintain data privacy [28]. In general, research should give more consideration to data management and security issues when implementing digital health tools, such as the control of user access or encryption of data, as these were not described in sufficient detail by all authors. Furthermore, the impact of the aforementioned data security methods was not explored by the study authors, and the user experience and perspectives on these methods would provide important insight on whether these methods, such as two-factor authentication or patient information stored in QR codes, worked in a practical manner.

### End-User experience

Eight out of eleven digital health tools included data on end-user perception and experience. The data collected from all studies was summarised and grouped into emerging themes, such as: usability, applicability, and feasibility. The data on end-user experience was collected through various methods, such as semi-structured interviews, questionnaires, and surveys, offering a comprehensive perspective on both the advantages and limitations of these digital tools, when applied in the resource limited setting, where skin NTDs are endemic.

### Usability

The theme of usability was prominent in most studies that offered insights into user experience. In this section, the term ‘usability’ has been defined as the extent to which the digital tools can be used by end-users to facilitate diagnosis of skin NTDs with effectiveness and efficiency, which includes factors such as intelligibility and system accessibility. Results from studies which conducted a semi-structured questionnaire have indicated that digital health tools were generally useful in facilitating diagnoses and were easy to operate [28,30], as all the various functions were well integrated, and the system was not ‘unnecessarily complex’ [28]. System-usability scores reported by the eSkinHealth app [28] indicate that although end-users initially felt the app required extensive learning, by week 12, they had adapted well and found it much easier to use. However, no further detail was given into the process of exactly how individuals became “adapted” to the use of the app over time. Semi-structured interviews conducted with health providers who used SkinApp as an aid in the diagnosis of skin NTDs reported that the mobile app provided information about skin NTD presentations in a more convenient and accessible way compared to other sources, such as traditional literature. The SkinApp was also deemed easy to use as its end-users were able to navigate through the app after only a short introduction into its functionalities, and that the narrative and illustrative content of the app was easy to understand [29]. Similarly, feedback from a user satisfaction survey of the telemedicine computer software in French Guiana had found that most users thought the system was useful for providing support in diagnosis and patient management, as well as minimised travel costs for the patient and decreased expenditure for the local healthcare system [37].

As per the survey conducted by Handa et al. [34], patients and health providers were mostly satisfied with the Hybrid tele-dermatology system, but providers expressed difficulty in assessing specific features of skin lesions, and establishing rapport with patients, over phone teleconsultation. Also, limited technological abilities of patients posed a barrier to successful teleconsultation, as well as system faults, such as incorrect patient details, which proved to be very time inefficient [34]. A survey completed by users of another telemedicine computer system reported many other system accessibility issues; the task of uploading images was time-consuming; request forms were too complex; and case management was not possible from a personal computer, preventing remote review of cases from locations other than one health facility which can be inconvenient for health providers [37].

Furthermore, adherence to the installation and routine use of Leishcare mobile app [30] proved challenging, as the results of semi-structured interviews revealed that most health workers who were receptive to the use of the mobile app had not even installed it after 6 and 12 months. As another study suggests [32], behaviour change can take time, and more qualitative research is required to examine the reasons behind health worker hesitance or low adherence towards these technological interventions in the health care setting.

In summary, according to feedback from end-users, the usability of digital health tools hinges on the degree of user-centered design and convenience they offer. Users appreciate clear and easily navigable system content, as well as straightforward tasks that avoid unnecessary complexity [28]. Although the Skin App provides workers with key information and diagnostic support for skin NTDs, adequate prior training is essential [29]. Training for the eSkinHealth app seemed overwhelming at first, but users who persisted with regular use found the system much easier to learn than expected [28]. On the other hand, transitioning to new technology proved challenging for Leishcare users, impacting routine use [30]. The reasons for poor adherence are likely complex, but, as indicated by other studies, users’ perceptions of system difficulty and convenience may play a significant role.

### Applicability

Another theme which emerged from the data on user-experience is applicability. In this section, the term applicability refers to the relevance and usefulness of the digital health tools in their specific role, for example, the digital tool acting as a diagnostic aid or a facilitator in communication between health workers, or health workers and patients. As per the semi-structured interviews conducted by Yotsu et al. [28], all nurses who used the eSkinHealth app were satisfied because they were able to contact remote dermatologists when dealing with difficult cases, and message them for support in making a diagnosis. However, the authors do not provide any further information on whether this provider-provider interaction was previously possible without the use of the eSkinHealth app, and they do not elaborate on how exactly the app contributed to, or changed, this interaction. Furthermore, the nurses expressed that the app helped them provide more precise and accurate diagnosis, as before the implementation of the app, they were not able to distinguish between different skin NTDs and reported all skin conditions as ‘dermatoses’. All dermatologists and program managers who participated in the study expressed that the eSkinHealth app can build capacity in peripheral areas by strengthening the capabilities of nurses and community health workers in screening, diagnosing, and treating skin diseases [28]. As another study points out, peripheral workers feel more confident in ascertaining diagnosis when specialists are available and accessible to provide answers to their doubts immediately [35]. Although the authors do not explicitly define what “peripheral” workers imply, based on the context of the study, they are likely trying to denote that these are health workers based in more rural or remote areas that do not have the same access to healthcare services and infrastructure as large urban centres. Furthermore, community health officers who used the MyTeleDoc mobile app [33] reported in the feedback questionnaire that the app helped them assess patients more thoroughly, because it ensured they did not miss any important steps. Qualitative feedback from users of the mobile-based LEARNS system and the Leishcare app reported that their digital tool had raised awareness around the skin NTD [30,35] or inspired them to consider it as a differential diagnosis [30].

Another survey highlighted an important issue experienced by users of a telemedicine computer software [37], which found that the quality or accuracy of the diagnosis can depend on the quality of the image and request sent by the peripheral health worker to the remote doctor. In over 44% of discordant cases, inadequate images or incomplete information provided by the CHO resulted in a discordant diagnosis. This suggests that health workers in the community should receive adequate training to improve their abilities in sending better quality requests to remote doctors, ultimately benefitting patients, and increasing the applicability of these digital interventions [33].

In summary, the applicability of the digital health tools in facilitating diagnosis depends on their efficacy in supporting the diagnosis of skin NTDs. This is demonstrated through methods such as providing clear instructions to peripheral workers, simplifying the diagnostic process, facilitating teleconsultations with patients, or enabling dialogue between peripheral health workers and specialist doctors. The quality of guidance obtained in this dialogue may depend on the end-user, such as the health workers’ proficiency in capturing a ‘high-quality’ photograph. Digital health tools prove to be more applicable when they empower health workers to deliver accurate and precise diagnoses for skin NTDs. Conversely, their relevance lessens when the quality or accuracy of diagnoses suffers, for instance, due to subpar information sent to remote specialists [28,33,37].

### Feasibility

The final theme, which was prominent across all studies, was the feasibility of digital health tools. In this section, feasibility refers to the aspects of practicality and suitability of the digital health tools in their sociotechnical context. Interviews with end-users have revealed that poor access to network and internet coverage remains to be a problem in rural and remote areas [28,30]. An offline adaptation can be useful in the context of skin NTDs, as many affected patients reside in areas where 4G or 5G internet access can be limited or non-existent. However, evidence shows that these offline adaptations still require further work, as mobile app users who utilised the offline function encountered several challenges when synchronising data or uploading images with a larger bandwidth [28].

Conversely, the eSkinHealth app, which can be used offline, was commended for its portability. Nurses and community health workers appreciated being able to take the tablet into the community and consult patients directly [28]. This demonstrates that a mobile application can enhance access to diagnosis and healthcare for patients in rural areas when supported by the appropriate infrastructure, such as providing tablet devices to health workers [28]. Users of the Guaral App [31] perceived the digital tool to have promising transferability, as most users who completed the feedback questionnaire stated that the app could be used in other rural communities in Colombia. However, there was no further detail provided on why end-users considered the Guaral App to be transferable.

One interview with a user of the mobile application Leishcare [30] revealed that despite the app being useful, it was more suitable for tertiary care, not primary care. They expressed that when they encountered a suspected case of leishmaniasis, they referred it to tertiary care, as they did not have access to rapid diagnostic tests at the primary level [30]. This highlights the technical infrastructure which is required to support the feasibility of these tools at the targeted health care level. As another study shows, mobile applications have shown to be useful screening and triage tools for confirmatory parasitological diagnosis of skin NTDs (such as cutaneous leishmaniasis), which is required before commencing treatment. In this way, the mobile app can help cut costs in the subsequent use of rapid diagnostic tests by health workers when they become available [31]. Yet, it becomes apparent that digital health tools can function effectively in this manner only when integrated into an infrastructure that sustains the continuum of care for skin NTDs.

In summary, the theme of feasibility identified many challenges to digital health tool implementation on the rural level, mainly linked to poor infrastructure. Poor network access in areas affected by skin NTDs necessitates adequate offline adaptations of existing digital health tools [28]. Similarly, a lack of resources, such as lack of rapid diagnostic tests at the primary care level, can pose problems for patients in rural areas, who because of this, may be forced to seek help in the tertiary care setting. As one study shows [33], rural patients would best benefit from digital health tools aimed at the community level, as they are more likely to face problems in care-seeking due to geographic and financial barriers. Enhancing the feasibility of digital health tools within community settings will contribute to ‘bridging the gap’ in access to dermatological care and help integrate these technological interventions so that they align with the local healthcare system. Nevertheless, those deploying digital health tools must carefully consider the advantages and drawbacks of integrating the digital tool at various tiers of the healthcare system. While incorporating the digital tool at a primary care level may appear to be a more financially viable and convenient option for patients, it could potentially give rise to other accessibility issues, particularly if rapid tests are not as readily available as they would be at the tertiary level.

## Discussion

The aim of this scoping review was to map the currently available evidence on digital health tools used to aid the diagnosis of skin NTDs. The findings of this study report on eleven different digital health tools which aid in the diagnosis or detection of skin NTDs, and describe the technical modality, function and application, policy considerations and end-user experience associated with these tools.

Although there are at least 10 skin NTDs prioritised by the WHO [4], most of the included studies only reported on digital health tools which aided in the diagnosis of leprosy, scabies or cutaneous leishmaniasis. Consequently, other skin NTDs, such as Buruli ulcer, lymphatic filariasis, onchocerciasis, and yaws, among others, have been underrepresented in the available data. Considering the co-endemicity of skin NTDs in affected areas, and their commonalities in producing cutaneous symptoms, further research is needed to develop digital health tools which integrate a wider range of skin NTDs, which would maximise the use of resources and improve their impact.

The included studies were conducted in only 10 different countries. Considering that NTDs are endemic in over 149 countries across the globe, this highlights the currently limited geographical scope of digital health tools which aid in the diagnosis and detection of skin NTDs. Testing these digital tools in more countries would expand the cultural and contextual evidence base for these technological interventions [39]. Although the results from one study conducted in Colombia have indicated that end-users considered the app to be “transferable” across other communities in Colombia, the study did not comment on why this was the case. Further studies are required to explore the transferability of these digital health tools not only across different countries but also across different regions within these countries.

The findings of this review highlighted the current lack of data on the diagnostic accuracy of digital health tools in aiding the diagnosis of skin NTDs. One of the studies assessed the sensitivity of the Guaral App in detecting true leishmaniasis cases, but characteristics such as negative predictive value and specificity were not assessed [31]. Similarly, the findings show that most studies did not address potential data security concerns or consider data protection measures. Although some of the includes studies describe data privacy measures such as “two-factor authentication” or “QR codes” for patient data security, more research is required to inform how end-users experienced these measures, and whether they were fit for purpose.

One of the included studies noted that users of the eSkinHealth app [28] initially found that the app required extensive learning but reported much greater ease of use by week 12. However, the data does not explain the reasons for this adaptation, or what specifically supports ease of use in this context. Therefore, implementation studies should examine the practices and experience of end-users who represent their target audience as closely as possible, exploring the factors that contribute to improved usability of digital health tools [28,37].

This review highlights the most common function of the digital health tools, which was to facilitate provider-to-provider tele-dermatology [30–33,35,37]. In general, digital health tools which facilitate interactions between peripheral workers and remote doctors or utilise digital diagnostic algorithms can be more beneficial for patients affected by skin NTDs in rural and resource poor areas, as the patient is not required to possess technological knowledge or equipment and can access remote dermatological care at the community level.

Countries where skin NTDs are endemic, such as those within Sub-Saharan Africa, suffer severe physician shortages with as few as ten physicians per 100,000 people, with no dermatologists in most areas [40]. The pronounced disparities in dermatologist availability between urban and rural areas exacerbate this gap in access to dermatological care [5]. One study from North India highlighted how physical barriers such as distance can be overcome by digital health tools, as patients registered for the tele-dermatology service were as far as 2000km from the facility [34]. In this way, digital health tools can help extend reach to patients for whom time and travel expenditure would render in-person health-care access economically infeasible [41].

As well as this, when combined with rapid diagnostic tests, digital health tools can help streamline the triage and treatment process of skin NTDs, which may be more cost-effective for local health facilities as rapid diagnostic tests will not be used on every patient [42]. However, one of the included studies has highlighted the lack of appropriate infrastructure and resources, such as rapid diagnostic tests, where digital health tools are deployed, which affects their impact and effectiveness [30]. Therefore, developers are encouraged to consider the resources available at the targeted health care level, so that the implementation of the digital health tool supports the continuum of care in that area.

The implementation of digital health tools to aid in the diagnosis of skin NTDs has brought additional challenges, one of which is picture quality. As one study points out, one may encounter several practical issues when attempting to take a good quality photograph; lighting, equipment, sizing references, and indeed the photography skills of the end-user are all required to capture the desired quality of image, which may be difficult to achieve without prior training or adequate facilities, such as a high-quality camera [43]. Therefore, users of digital health tools that involve sending photographs to remote physicians should receive sufficient training and resources to enhance the quality of referrals, thereby assisting physicians in making accurate diagnoses.

Transitioning to digital methods in healthcare delivery can be overwhelming for both healthcare providers and patients. This issue was highlighted by the Leishcare app [30] which reported low adherence to the installation and routine use of the app among health workers. One way to break down these barriers is to provide patients and health providers adequate assistance in the usage of new digital tools and incorporate their feedback into the continuous development of these technologies [44]. For example, studies which explore user perception of mHealth technologies have highlighted that, when digital tools are perceived to be difficult to operate, health workers may become frustrated and feel reluctant to use the technology [45]. Therefore, additional qualitative studies are needed to inform the experiences of end-users with digital health tools used for diagnosing skin NTDs, to ensure these tools meet the needs of health workers effectively.

Furthermore, this review has highlighted the complex challenge of introducing secure digital health tools in regions affected by skin NTDs. This complexity arises from inadequate infrastructure prevalent in countries lacking a robust foundation in the legal aspects of medical technology. One study from India illustrates the lack of guidance on patient data storage, leaving the responsibility for data security in the hands of physicians [34]. Notably, gaps in data protection systems in India re-surfaced during the COVID-19 pandemic, which led to significant health data breaches and leaked confidential patient information during this period. Notably, the lack of technical and legal infrastructure, such as those in the example above, may instil reluctance among health workers to utilize third-party applications on mobile devices, tablets, or computers. Consequently, implementing digital health tools in regions lacking a robust legal infrastructure may compromise the safety and feasibility of these interventions [46].

Lastly, the findings of this study have shown that some digital health tools may be more applicable for certain skin NTDs than others. Some of the included studies focused on skin diseases other than skin NTDs, but despite this, their digital tool was able to diagnose scabies due its easily distinguishable clinical features, especially on clinical photographs [38]. However, authors of another study reported difficulty in diagnosing subcutaneous conditions remotely through regular photographs, where the lesion affects deeper tissues. This suggests digital health tools which utilise solely photographic images are useful for superficial lesions but may be less useful for subcutaneous skin conditions [28]. As the latter indicates, different skin NTDs may present in clinically different ways, and the efficacy of a digital tool in assisting the diagnosis of a skin NTD may hinge on the clinical manifestation of the skin condition. Developers of digital health tools should consider adapting technical features of existing tools, so that they can detect superficial and deeper skin lesions with equal effectiveness.

In summary, this review has highlighted the potential of digital health tools in improving the diagnosis and detection of skin NTDs. Digital health tools which are portable and include offline adaptations can help expand dermatological care to rural and resource-poor communities affected by skin NTDs. However, digital health tools should be deployed at the appropriate health care level which best supports the continuum of care, aligning with the infrastructure available at that level. Further research should be conducted to determine the diagnostic accuracy of digital health tools, as well as address data security issues to protect confidentiality. Also, qualitative studies on user-experience are necessary to inform the usability of digital health tools, exploring the factors which contribute to their successful adaptation and adherence to routine use. Overall, more studies across different countries are needed to inform the transferability of these tools across different contexts and what additional resources or adaptations may be required to align the technology with specific health care needs and practices.

## Limitations

This scoping review allowed the comparison and contrast of the findings of several studies which utilised different digital tool which aided the diagnosis of skin NTDs. However, scoping reviews are not designed to determine methodological quality of included studies; thus, it has not been assessed in this review [20]. Some of the included studies have generated memory bias [37] and observer bias [33] in their methodology and therefore the findings of this review should be interpreted with caution. Furthermore, this review focused on the skin NTDs which have been prioritised by the WHO [4]. Consequently, other NTDs exhibiting cutaneous manifestations, like Human African Trypanosomiasis [47], which could potentially be addressed by digital interventions, were beyond the scope of this review. However, future studies are encouraged to extend the scope of research beyond the ten skin NTDs prioritised by the WHO and explore how other NTDs with cutaneous manifestations could also possibly benefit from these technological interventions.

## Conclusion

The studies included in this review have highlighted the potential of digital health tools in facilitating the diagnosis of skin NTDs and helping decrease the interval between symptom onset and diagnosis in resource limited settings. However, the review has demonstrated the current lack of evidence on the diagnostic accuracy of existing digital health tools and the need for high-quality studies which will evaluate the performance of these interventions before implementation. Similarly, most included studies did not discuss potential data privacy and management concerns, which poses many ethical questions with regards to the safety of existing digital health tools. This review also highlights that most of the existing literature has focused on skin NTDs such as leprosy, scabies, or cutaneous leishmaniasis, which in comparison, leaves many skin NTDs such as buruli ulcer, yaws, onchocerciasis or mycetoma under-represented in this field. Also, only two of the included studies report on five or more skin NTDs. Considering the recent push by the WHO to design integrated interventions which have an impact on several NTDs at any given time [4], the current evidence leaves much room for improvement in expanding the scope of current digital health tools and maximising their impact. Based on the existing evidence gathered by this scoping review, several recommendations have been made for the future development of digital health tools for the diagnosis of skin NTDs, highlighting areas for further research in this field. Testing these tools in a wider range of countries and including other neglected tropical diseases with cutaneous manifestations will provide a more comprehensive understanding of their feasibility and potential in improving the diagnosis of skin NTDs on a global scale.

## Recommendations

The findings of this scoping review have highlighted the perceived benefits of using digital health tools adapted for off-line use to facilitate the diagnosis of skin NTDs in rural and resource limited settings. However, developers are encouraged to further fine-tune these adaptations, given the existing issues with synchronising data or uploading images with a larger bandwidth.

Furthermore, developers and health providers should examine the availability of necessary resources, such as confirmatory laboratory rapid tests, at the relevant healthcare level, so that these can be deployed alongside digital health tools to prevent treatment delays and patient anxiety. Alongside this, developers should provide the necessary training programs and technical support for end-users which are crucial for the long-term sustainability of digital health tools. They should collaborate with local health institutions to evaluate the tools’ compatibility with local health information systems and their potential to enhance or disrupt current practices.

Further research is required to address the data security issues associated with digital health tools, so that they comply with international standards and best practices for data protection. Policymakers in regions affected by skin NTDs are urged to enforce legislation that mandates strict data protection requirements for digital health tools.

Future studies should prioritise assessing the diagnostic accuracy of digital health tools, to provide evidence on their effectiveness in facilitating accurate skin NTD diagnoses. Moreover, this scoping review has highlighted issues around usability and low adherence towards the routine use of digital health tools. Future research should provide more insight into the challenges of aligning digital health tools with the needs of health workers and the specific contexts in which they must operate. Conducting in-depth interviews with end-users can inform the strategies to address these challenges.

Digital health tools which aid the detection of more than one skin NTD at any given time can help pave the way towards their integrated management, as envisioned by the WHO in the 2021–2030 Strategic Framework [4]. Most of the studies included in this review have been able to detect only one skin NTD, such as leprosy, scabies or cutaneous leishmaniasis. Developers are encouraged to expand the disease scope of digital health tools to increase their impact and effectiveness.

Future studies are encouraged to build on this scoping review to further contextualise the current digital health tools used to aid in the diagnosis of skin NTDs. The included studies in this review contain data from only 10 countries, thus, the use of digital health tools for this purpose should be tested in more countries across the globe, to provide more evidence on their feasibility in different contexts.

As well as this, future studies are encouraged to look beyond the scope of the 10 skin NTDs prioritised by the WHO and consider including other NTDs which display cutaneous manifestations, such as Human African Trypanosomiasis, to determine the potential of digital health tools in addressing these equally neglected conditions. Overall, these efforts can help extend the benefits of digital health technologies to a larger patient population, thus enhancing the overall impact of these innovations in global health.

## Supporting information

S1 Checklist PRISMA-ScR Checklist. (DOCX)

S1 Table Search Strategy. (DOCX)

S2 Table Key features of digital health tools. (DOCX)

S3 Table End-user experience and challenges to implementation. (DOCX)
