# Supplementary material for: Diagnosing skin neglected tropical diseases with the aid of digital health tools: A scoping review
Source: PLOS Digit Health. 2024 Oct 7;3(10):e0000629. doi: 10.1371/journal.pdig.0000629 (PMC11458012; doi:10.1371/journal.pdig.0000629)
Supplement: S1 Table — (DOCX) [file pdig.0000629.s002.docx]

**S1 Table** Search Strategy

Final search: 24.07.2023

| Database | Search String | Results |
| --- | --- | --- |
| Pubmed | ("leprosy"[MeSH Terms] OR "elephantiasis, filarial"[MeSH Terms] OR "onchocerciasis"[MeSH Terms] OR "yaws"[MeSH Terms] OR "scabies"[MeSH Terms] OR "mycetoma"[MeSH Terms] OR "buruli ulcer"[MeSH Terms] OR "leishmaniasis"[MeSH Terms] OR "leprosy"[Title/Abstract] OR "elephantiasis"[Title/Abstract] OR "onchocerciasis"[Title/Abstract] OR "yaws"[Title/Abstract] OR "scabies"[Title/Abstract] OR "mycetoma"[Title/Abstract] OR "buruli ulcer"[Title/Abstract] OR "leishmaniasis"[Title/Abstract] OR (("skin"[Title/Abstract] OR "dermatology*"[Title/Abstract] OR "cutaneous"[Title/Abstract] OR "Skin Diseases"[MeSH Terms] OR "skin disease*"[Title/Abstract]) AND ("neglected tropical disease*"[Title/Abstract] OR "NTDs"[Title/Abstract] OR (("Neglected Diseases"[MeSH Terms] OR "neglected"[Title/Abstract]) AND ("Tropical Medicine"[MeSH Terms] OR "tropical"[Title/Abstract]))))) AND ("diagnos*"[Title/Abstract] OR "diagnosis"[MeSH Terms] OR "early diagnosis"[MeSH Terms] OR "diagnostic services"[MeSH Terms] OR "referral and consultation"[MeSH Terms] OR "disease notification"[MeSH Terms]) AND ("telemedicine"[MeSH Terms] OR "cell phone"[MeSH Terms] OR "remote consultation"[MeSH Terms] OR "text messaging"[MeSH Terms] OR "mobile applications"[MeSH Terms] OR "SMS"[Title/Abstract] OR "mobile health"[Title/Abstract] OR "mhealth"[Title/Abstract] OR "telemedicine"[Title/Abstract] OR "ehealth"[Title/Abstract] OR "mobile phone application"[Title/Abstract] OR "teledermatology"[Title/Abstract] OR "telemedicine"[Title/Abstract] OR "remote consultation"[Title/Abstract] OR "technology"[Title/Abstract] OR "distance consultation"[Title/Abstract]) | 176 |
| Embase | (exp leprosy/ or exp elephantiasis/ or exp lymphatic filariasis/ or exp onchocerciasis/ or exp yaws/ or exp scabies/ or exp mycetoma/ or exp Buruli ulcer/ or exp leishmaniasis/ or (leprosy or elephantiasis or 'lymphatic filariasis*' or onchocerciasis or yaws or scabies or mycetoma or 'buruli ulcer*' or leishmaniasis).ti,ab,kf. or ((exp skin disease/ or exp skin/ or exp dermatology/ or (skin or cutaneous or dermatology).ti,ab,kf.) and (('neglected tropical disease* ' or ntd or ntds).ti,ab,kf. or ((exp neglected disease/ or neglected.ti,ab,kf.) and (exp tropical medicine/ or tropical.ti,ab,kf.))))) and (exp diagnosis/ or exp early diagnosis/ or exp patient referral/ or exp disease notification/ or diagnos*.ti,ab,kf.) and (exp telemedicine/ or exp mobile phone/ or exp teleconsultation/ or exp text messaging/ or exp mobile application/ or (SMS or 'mobile health' or mhealth or telemedicine or ehealth or 'mobile phone application' or 'teledermatology' or 'remote consultation' or 'distance consultation' or technology).ti,ab,kf.) | 313 |
| SCOPUS | ( TITLE-ABS-KEY ( "leprosy" OR "elephantiasis" OR "lymphatic filariasis" OR "onchocerciasis" OR "yaws" OR "scabies" OR "mycetoma" OR "buruli ulcer" OR "leishmaniasis" OR ( ( "skin" OR "skin disease" OR "dermatology" OR "cutaneous" ) AND ( "neglected tropical disease" OR "NTD" OR ( "neglected" AND "tropical" ) ) ) ) ) AND ( TITLE-ABS-KEY ( diagnos* ) ) AND ( TITLE-ABS-KEY ( "SMS" OR "mobile health" OR "mhealth" OR "telemedicine" OR "ehealth" OR "mobile phone application" OR "teledermatology" OR "telemedicine" OR "remote consultation" OR "distance consultation" ) ) | 62 |
