# Supplementary material for: Diagnosing skin neglected tropical diseases with the aid of digital health tools: A scoping review
Source: PLOS Digit Health. 2024 Oct 7;3(10):e0000629. doi: 10.1371/journal.pdig.0000629 (PMC11458012; doi:10.1371/journal.pdig.0000629)
Supplement: S2 Table — (DOCX) [file pdig.0000629.s003.docx]

**S2 Table** Key features of digital health tools

| Digital tool, 1st Author | Functionality | Application | Technical modality | Policy considerations | |
| --- | --- | --- | --- | --- | --- |
| **Robotic teledermatopathology**  (36) | - Consultation - Diagnosis - Management | - Assists in establishing a definitive diagnosis of several diseases, including leprosy and scabies - Applied in resource limited setting (Botswana) | - Consultation via Robotic microscope, remote review - Email store-and-forward used to send additional photographs | Data security/management issues not discussed. | |
| **LEARNS**  (35) | - Consultation - Diagnosis - Management | - Assists peripheral healthcare workers in identifying and diagnosing cases of leprosy. - Complements leprosy control and monitoring programmes - Applied in endemic area (Iloilo, Philippines) | - Mobile phone teleconsultation - Images of the skin lesion and patient information are sent through the provider’s mobile phone. - short-message service (SMS) is also accepted when providers have no camera | Standardized de-identified patient information was used to maintain confidentiality | |
| **NLR SkinApp**  (29) | - Consultation - Diagnosis - Management - Mentoring | - Assists community health workers in making decisions regarding the diagnosis and treatment of several skin NTDs (such as buruli ulcer, cutaneous leishmaniasis, leprosy, lymphatic filariasis, mycetoma, onchocerciasis, podoconiosis, scabies, yaws) - Applied in rural and resource limited settings | 2^nd^ Version of Skin App   - Mobile app - Uses an algorithm to support diagnosis. - Available on Android as well as iOS operating systems - Once downloaded can be used offline. - Available in English and Portuguese - Teledermatology option/feedback button | Data security/management issues not discussed | |
| **Telemedicine computer software**  (37) | - Consultation - Diagnosis - Management - Referral | - Allows non-specialist health workers to send cases through a telemedicine platform reviewed by a dermatologist to provide a diagnosis. - Skin NTDs such as scabies, leprosy, cutaneous leishmaniasis, tungiasis were diagnosed via the telemedicine platform. - Applied in remote/rural areas of French Guiana | - Computer with access to telemedicine software, with a digital camera - Internet connection is required. - Photos can be attached to the referral request form | Network is secure and is appropriate for the sharing of medical data. | |
| **Real time face-to-face tele dermatology**  (38) | - Consultation - Diagnosis - Management - Monitoring (follow-up) | - Facilitates a face-to-face real time consultation between patient and dermatologist. - Features such as scaling and burrowing of the skin consistent with Scabies were recognised and diagnosed. - Applied in rural areas (Taitung county) | - Intouch lite® software facilitates the two-way audio and video connection (synchronous) - Can be installed on any PC or tablet device. - Requires internet connection. - Possibility of pairing the software with a USB digital handheld diagnostic device (dermoscope) with field of view (10mm) which allows detailed assessment of skin in 2M pixel. - Images and videos can be captured for further investigation. | - Data security/management issues not discussed | |
| **Leishcare app**  (30) | - Consultation - Diagnosis - Management - Monitoring | - Allows health workers to register patient data and clinical information during a consultation and share this data with physicians to facilitate diagnosis. - Aids prognostic assessment and follow-up of patients - Concerns only visceral and tegumentary leishmaniasis (skin/mucosal) - Applied in rural and endemic areas of Brazil | - Mobile application for smartphones with Android operating system - Internet access is required for data sharing. - Data can be shared through e-mail, Bluetooth or applications that allow data sharing (on which the Leishcare app is installed) | Data security/management issues not discussed | |
| **Guaral/Leishmaniasis app**  (31) | - Diagnosis - Referral | - Facilitates community based presumptive diagnosis of cutaneous leishmaniasis. - The App is used to screen patients for components of a previously validated clinical prediction rule, if the patient scores 7/10 or higher, they are referred to a health provider for diagnosis/treatment. - Applied in endemic, resource limited setting in Colombia (with limited road access, poor or none 3G internet coverage) | - Mobile app with Android operating system - Tested using an inexpensive and locally available smartphone. - A corresponding web-based application (SND platform) stores data obtained through the mobile app. - Data obtained through the mobile app can be stored off-line and later transferred to the SND platform when internet connectivity is re-established.   Offline usage benefit=internet connectivity/network coverage poor in some areas  Beneficial use of Android=widespread availability on low-cost smartphones. App was tested on a low-cost, locally available smartphone Motorola Moto G | Security features:   - Strong encryption - Two factor authentication | |
| **Hybrid teledermatology**  (34) | - Consultation - Diagnosis - Management - Monitoring (follow-up) | - This smart phone based teledermatology model was used to conduct teleconsults with patients and additional images/information was sent through the mobile application Whatsapp Messenger - Diagnoses include leprosy and ectoparasitic infection (among others) - Applied in a tertiary care centre during the COVID-19 pandemic (North India) | - Hybrid of store-and-forward (S&F) and real-time teledermatology (RTTD) - Phone calls made on an Android smartphone with 4G internet connection. - Supplemented by Whatsapp messenger mobile application | Maintaining records is the responsibility of the physician, there is a lack of guidance or clarity regarding the duration of data storage in India | |
| **Teledermatology via Viber® mobile app**  (32) | - Consultation - Diagnosis - Management | - Used to conduct photo consultations with dermatologists to confirm probable diagnosis of cutaneous leishmaniasis which allowed appropriate treatment to be commenced. - Applied in a remote district of Nepal | - Store-and-forward (S&F) teledermatology via Viber**®** mobile application | Data security/management issues not discussed | |
| **eSkinHealth app**  (28) | - Consultation - Diagnosis - Monitoring (follow-up) - Management | - Used for on-site and remote diagnosis and monitoring of skin diseases. - Adapted for skin type IV and darker and use in LMICs. - Provides support in clinical decision making and mapping of skin diseases. - Can be used for skin NTDs such as buruli ulcer, leprosy, lymphatic filariasis, scabies, yaws. - Applied in rural areas of Côte d’Ivoire with co-endemicity of several skin NTDs | - A mobile application currently available for Android tablets with an operating system version above 9.0. - Contains features as such as: Patient ID and demographics, symptom list, photo list, evolution list. - App is paired with a web-based platform for facilitating a teleconsultation with remote dermatologist for support in diagnosis, treatment, and counselling. - Constant internet connection is not required - necessary information can be recorded offline. - Offline data collected by the app can be synchronised to the platform via database server once internet access is re-established. | - Patient information security using QR codes – information is portable and secure. - Data is stored using the Simple Storage Service (S3) of the Amazon Web Service server. The server is secure, regulated and encrypted, with continuous 24hr data back-up. - Access to data in the app is regulated through user levels. | |
| **MyTeleDoc app**  (33) | - Consultation - Diagnosis - Management - Referral | - Used for facilitating tele consults between community health officers (CHO) and remote doctors. - CHO can send cases through the app and receive diagnosis and triage decision from a remote doctor. - Used to diagnose and manage a variety of conditions including Scabies. - Applied in rural low-income areas of Morbi district in Gujarat, India | - MyTeleDoc app is an open-source telemedicine software. - Consists of a mobile application and a digital assistant Ayu, which guides the CHO’s history taking - The doctor can conduct video/audio tele consults with CHO or the patient (synchronous) or may provide the diagnosis and management decisions without speaking to the patient or CHO (asynchronous). | Data security/management issues of the app are not mentioned. |  |

*In chronological order
