# Supplementary material for: Diagnosing skin neglected tropical diseases with the aid of digital health tools: A scoping review
Source: PLOS Digit Health. 2024 Oct 7;3(10):e0000629. doi: 10.1371/journal.pdig.0000629 (PMC11458012; doi:10.1371/journal.pdig.0000629)
Supplement: S3 Table — (DOCX) [file pdig.0000629.s004.docx]

**S3 Table** End user-experience and challenges to implementation

| Digital tool and reference | End-user experience (provider and patient feedback) | Challenges/weaknesses identified |
| --- | --- | --- |
| **Robotic teledermatopathology** (36) | None described | None described |
| **LEARNS** (35) | Provider feedback   - The system is easy to use. - Raises awareness for leprosy. - Increases confidence of peripheral healthcare workers in clinical diagnosis | None described |
| **NLR SkinApp**  (29) | Provider feedback   - The app is more easily accessible than literature or books. - It is easy to operate. - Illustrative/narrative content was clear to understand. | None described. |
| **Telemedicine computer software**  (37) | Provider feedback:   - Majority of users found the system to be useful for providing diagnosis support improving patient management, minimising travel costs for the patient and decreasing expenses for the healthcare system. | - Only 60% of images were judged as good quality. - Only 46% users reported 95% diagnostic certainty. (Possibly due to poor image quality) - Case management is not possible from a distance. - Poor access to network and mobile internet coverage in many areas in French Guiana. - importing and sending images is time consuming, request forms are too complex |
| **Real time face-to-face tele dermatology** (38) | None described. | None described. |
| **Leishcare**  (30) | Provider feedback   - the app is useful, easy to use and accessible with quick feedback. - the app could be more useful in the tertiary care setting, as there is insufficient support and infrastructure within the primary care setting. - the app raises awareness and encourages health providers to consider leishmaniasis as a differential diagnosis | - Low adherence to installation and use of the app |
| **Guaral/Leishmaniasis app**  (31) | Provider feedback   - App was easy to use. - The app allows for rapid identification of CL lesions. - The app could be used in other rural communities in Colombia. | None described. |
| **Hybrid Teledermatology**  (34) | Patient feedback:  Most patients were satisfied with the tele dermatology service.  Provider feedback:   - Convenience, ease of communication - Difficulty in assessing morphological/topographical features of skin lesions. - Difficulty in establishing rapport with patients over the phone. - Technical issues (internet connectivity) | - While 7530 patients were registered for teleconsultation, only 6125 tele consults were able to be provided. - Challenges identified to teleconsultation were incorrect contact details, duplicate cases, problems with connectivity, and poor technological ability of the patient. |
| **Teledermatology via Viber® mobile app**  (32) | None described | None described |
| **eSkinHealth app**  (28) | Provider feedback:   - System usability scores increased after 12 weeks (adjustment time) - Over synchronisation of data in remote and rural areas of poor network and internet connectivity - Need for a flash function for night-time use. - One nurse noted the image quality was poorer compared to a smartphone. - Nurses who used the app were satisfied because they can contact dermatologists for help. - Most CHWs were satisfied with the app and tablet as it gave them self-confidence in their work. - The app is portable – nurses and CHWs they were able to consult patients in the community. - App has potential to build capacity for nurses and CHWs in peripheral areas. - All nurses expressed that the app helped them provide more accurate diagnosis. | - Despite the app being capable of offline usage, challenges were still faced in rural areas with poor network/internet connectivity when synchronising data and uploading images with a larger bandwidth. |
| **MyTeleDocApp**  (33) | - The app helped doctors work more efficiently. - CHO’s expressed the app helped them to assess patients more thoroughly | - The quality/accuracy of the diagnosis may depend on the abilities and skills of the CHO in using the app and digital assistant. In atleast (44%) discordant cases, incorrect, incomplete information provided by the CHO resulted in a discordant diagnosis. |

*In chronological order
